# Editorial: Genetics and epigenetics: Plausible role in development of climate resilient crops

**DOI:** 10.3389/fgene.2023.1165843

**Published:** 2023-03-10

**Authors:** Abhishek Bohra, Vijay Gahlaut, Dragan Perovic, Rajeev K. Varshney

**Affiliations:** ^1^ State Agricultural Biotechnology Centre, Centre for Crop and Food Innovation, Food Futures Institute, Murdoch University, Murdoch, WA, Australia; ^2^ University Centre for Research and Development, Chandigarh University, Mohali, Punjab, India; ^3^ Federal Research Centre for Cultivated Plants, Julius Kuehn Institute, Institute for Resistance Research and Stress Tolerance, Quedlinburg, Germany

**Keywords:** candidate genes, climate-resilient crops, genetics, epigenetics, stress response

Rising weather extremes and evolving pest and pathogen dynamics associated with climate change exert profound negative impacts on global crop production. Plants are sessile organisms, and intrinsic mechanisms enable them to respond to a variety of challenges posed by stressful conditions. Technological advances, especially in development of new high-throughput sequencing technologies, in recent years have contributed greatly to improving our ability to understand the genetic and epigenetic changes occurring in plants, particularly in response to stresses. This Research Topic on “*Genetics and epigenetics: Plausible role in development of climate resilient crops*” presents 16 articles from leading experts in this field. We summarize key highlights of these articles in this editorial.

The epigenetic modifications are defined as heritable changes occurring beyond DNA sequences. In this context, Saeed et al. reviewed the recent advances to analyse epigenetic changes when plants are exposed to abiotic and abiotic stress conditions. The article underscores emerging techniques to analyse genome-wide epigenetic modifications such as sodium bisulphite sequencing, methylated DNA immunoprecipitation (MeDIP) and integration of these with evolving sequencing platforms including the next and third generation platforms. The authors also discuss how the improved methods can contribute to separate the contributions of epigenetic modifications to phenotypes from other source of variations, e.g., sequence variations. The article also explores the possibilities to implement modern genome editing tools such as engineered endonucleases to analyse the epigenetic changes for improving plant stress response. In another article, Singroha et al. reviewed epigenetic dynamics and resulting alterations in gene expression reported in plants under salt-stressed scenarios. The article discusses the influence of salt stress on plant epigenetic machinery that involves DNA methylation, histone modifications, histone variants and non-coding RNA molecules including the long non-coding RNAs and microRNAs. For a better understanding of the epigenetic variations; Guarino et al. define the “epigenetic code” and recommend translating this code into “epigenetic syntax” for developing new crops carrying climate adaptation traits. The article highlights challenges that have cropped up as the field of epigenetics evolves. The authors call for modern sequencing technologies, optimized breeding strategies, standardized workflows for data analysis and integration of multi-omics data, to efficiently exploit epigenetic variation for crop improvement. DNA methylation is the most common epigenetic phenomenon leading to transcriptional silencing of genes and enabled by four different methyltransferases (MTases) in three sequence contexts: CG, CHG and CHH. Equally important to DNA methylation homeostasis are demethylases (dMTases) that remain instrumental to C methylation removal. Gahlaut et al. identified and characterized 12 dMTase in wheat by analysing the genome sequence information. The identified genes belonged to two subfamilies: DEMETER-LIKE (DML) and REPRESSOR OF SILENCING1 (ROS1), and mapped onto nine chromosomes. The study suggested a higher number of dMTase genes in wheat than other plant species, which are reported to vary between 2 and 10 in different plant species. Using these genes, the study demonstrated phylogenetic relationships, gene structure, regulatory function, nuclear localization signals (NLS) and DNA marker development. Analysis of gene expression patterns indicated a role for these genes in tolerance to heat stress in wheat.

Transcription factors (TFs) are known to regulate the cellular processes. AP2/ERF family represents the largest group of TFs among the 60 different TF families discovered so far in plants (Joshi et al., 2016). The availability of whole genome sequence has facilitated genome-wide identification and characterization TFs in plants. Since the role of the AP2/ERF family was demonstrated in flower development in Arabidopsis (Jofuku et al., 1994), growing literature has provided evidence in support of involvement of the AP2/ERF family in plant growth and development and stress responsiveness. Cui et al. used genome sequence information of Tifrunner, a popular groundnut (*Arachis hypogaea*) variety and identified 185 AP2/ERF family genes belonging to five sub-families e.g., AP2 (59), ERF (76), DREB (41), RAV (4), and Soloist (5). The study examined the phylogenetic relations and intron-exon structure and demonstrated that the identified genes are unevenly distributed among 20 chromosomes. Further analysis of the orthologous gene pairs between *A. hypogaea, Medicago truncatula* and *Glycine max* predicted the divergence times between *A. hypogaea* and *M. truncatula* (64.7 Mya), and *A. hypogaea* and *Glycine max* (66.44 Mya). Differential expression of 35 selected AP2/ERF family genes supported their roles in abiotic stress response. A similar survey of wheat genome sequence for receptor-like kinase (RLK) gene family led to the identification and characterization of 15 *TaRPK* genes (Rahim et al.). Gene expression analysis of tolerant (Pakistan 13 and Galaxy) and susceptible (Shafaq) varieties suggested *TaRPK*’s participation in drought tolerance in wheat (*Triticum aestivum*). Also, the cis-regulatory element (CRE) prediction showed abundance of drought-responsive elements for binding in the promoter regions.

Next-generation sequencing (NGS) has revolutionized the field of functional genomics by facilitating detailed inquiries into regulation of gene expression. Singh et al. employed Illumina technology to construct a *de novo* transcriptome assembly of an allohexaploid *Brassica* (AABBSS), comprising 486,066 transcripts. The novel allohexaploid has resulted from somatic hybridization between an amphidiploid *Brassica juncea* (AABB) and diploid *Sinapsis alba* (SS). In wheat, an NGS-based profiling of flag leaf transcriptome of cultivar KRL 3–4 increased understanding of its high level of tolerance against sodicity (Prasad et al.). The analysis revealed a set of 1,980 genes that respond differentially to sodicity stress. Authors provide a list of 18 candidate genes and 39 SNPs potentially associated with the sodicity-responsive genes. Similarly, RNA-Seq analysis of the root and shoot transcriptomes of PBW677 (nitrogen-efficient) and PBW703 (N-inefficient) revealed differential expression of 2,406 genes between the two contrasting genotypes. Nitrogen use efficiency (NUE) is an important breeding target in wheat improvement, owing to the increasing environmental risks associated with greater use of nitrogen fertilisers. The higher nitrogen efficiency of PBW 677 could be explained by expression abundance of the genes belonging to nitrogen metabolism and protein kinases.

By using the high-throughput chromosome conformation capture (Hi-C) technique, Yadav et al. examined the impact on stress conditions and hormone application in the dynamics of chromatin interactions in *Arabidopsis*. The study showed that the changes in chromatin interactions from stress conditions did not alter the expression profiles of the interacting genes. Interacting genes were found to be enriched in the heterochromatic regions and likely to belong to the same epigenetic state.

An article by Kumar et al. provides an overview of the Indian Wheat Genomics Initiative aimed at harnessing the untapped genetic potential of the wheat germplasm collection held at National genebank of ICAR-National Bureau of Plant Genetic Resources (NBPGR), India. NBPGR, India houses more than 31,000 wheat accessions from 51 species, representing the largest germplasm collection of wheat in Asia. Major challenges that limit the progress of Indian wheat improvement programs include a range of biotic (rust, Karnal bunt, Fusarium head blight, spot blotch, powdery mildew and other pathogens/pests) and abiotic (drought, heat and salinity) stresses. Other important breeding targets of Indian wheat improvement programs include nutrition and quality traits and nutrient use efficiency. Chandana et al. discuss the role of epigenomics for developing improved chickpea that can withstand agricultural conditions exposed to a variety of biotic and abiotic stresses. While citing recent research demonstrating the role of epigenetics stress response in chickpea and other legume crops, the authors advocate for embracing newer technologies to profile epigenomic variations and multi-disciplinary approaches to enable their efficient deployment in chickpea improvement programs by combining multi-omics science and targeted epigenetic manipulation.

Rising temperature extremities negatively impact normal growth and development of plants. In this context, low temperature leading to chilling stress (0°C and 15°C) and freezing stress (<0°C) remains a greater concern for sustaining global crop production (Jha et al., 2017). Satyakam et al. explain genetic, epigenetic, physiological and biochemical basis of cold adaptation in plants, with emphasis on cold acclimation. The authors outline strategies to improve cold tolerance in plant, such as targeted manipulation of anti-freeze proteins.

Identification and manipulation of genomic loci associated with climate adaptation is crucial for developing future crops. Artificially created populations (biparental/multiparent population) and diverse collections have facilitated understanding the genetic architectures of complex plant traits for (Bohra et al., 2020; Varshney et al., 2021a; Varshney et al., 2021b). A QTL for high grain yield and harvest index on chromosome 2BS was transferred from wild emmer (*Triticum turgidum* subsp. dicoccoides) to durum wheat cultivar Uzan (*Triticum turgidum* subsp. *durum*) following fine mapping and marker-assisted backcrossing approach (Deblieck et al.). The study shows implications of a high-throughput phenotyping platform for monitoring plant response under stress conditions. The resulting set of 2B introgression lines carrying QTL for culm length and kernel number would serve as a good resource for breeding wheat for water limiting conditions. A popular alternative to biparental QTL mapping is genome-wide association studies (GWAS) which was implemented by Dharmateja et al. for identification of genomic loci associated with phosphorus use efficiency. The study identified 45 QTL in P-limiting conditions in 158 wheat genotypes including popular varieties and advanced breeding lines analysed with Axiom BreedWheat 35 K genotyping array. The need for identification of a significant set of genetic markers to inform selection decisions is bypassed by genomic selection (Crossa et al., 2017; Bhat et al., 2021). In this Research Topic, the application of genomic selection (GS) was discussed in improving stress tolerance and quality traits of various crop species including cereals, pulses, oilseeds and horticultural crops (Budhlakoti et al.). The authors compare different genomic prediction models and outline key considerations related to genomic prediction accuracies in plant breeding programs. The cutting-edge genomic tools including whole genome sequence, candidate or causative genes, gene-trait associations or associated DNA markers, and genomic prediction models reported in these studies would be helpful for designing breeding strategies to obtain modern cultivars having new traits enabling cultivation in target production environments. As shown by Kumar et al., the multi-disciplinary initiatives will leverage of modern genomic resources and methods like GWAS and GS, thus paving the way for “Genebank genomics” and use of diverse germplasm collections for sustainable crop improvement.
